# Ten years of Chagas disease research: Looking back to achievements, looking ahead to challenges

**DOI:** 10.1371/journal.pntd.0005422

**Published:** 2017-04-20

**Authors:** Eric Dumonteil, Claudia Herrera

**Affiliations:** Department of Tropical Medicine, Vector-Borne Infectious Disease Research Center, School of Public Health and Tropical Medicine, Tulane University, New Orleans, Louisiana, United States of America; Yale School of Public Health, UNITED STATES

Chagas disease, caused by the protozoan parasite *Trypanosoma cruzi*, is a neglected tropical disease (NTD) with a high disease burden in the Americas. It is transmitted primarily by hematophagous triatomine bugs, but alternate transmission routes such as congenital, oral, and transfusional transmission are becoming more relevant in many regions. After a short acute phase, infected patients enter an asymptomatic chronic phase, which can become symptomatic in 20% to 40% of the cases, characterized by a chronic chagasic cardiomyopathy and less frequently by a digestive form of the disease. Current control is mostly focused on vector control with indoor spraying of residual pyrethroids and to a lesser extent with housing improvement. Treatment of infected patients remains challenging due to the limited efficacy of the two available drugs during the chronic phase and their side effects but also because of limited access to treatment for patients.

Over the past ten years, *PLOS Neglected Tropical Diseases* has been a key journal for the diffusion of some of the major achievements to better understand and control Chagas disease. With a total of 390 published studies during that time, Chagas disease research represented about 8% of the published material by the journal. Some of the key issues addressed have been related to the evaluation of disease burden, improvement in serological and molecular diagnostics, drug development, patient care, and vector control (Table 1).

**Table 1:** Challenges and research priorities for Chagas disease.

Area	Challenge	Priority
**Epidemiology**	• Discordance among serological tests • Limited information of circulating parasite genotypes • Improvement in disease surveillance	• Identification of novel antigens and development of new diagnostic tests • Development of more sensitive genotyping tools for molecular epidemiology • Improvement in the evaluation of disease burden
**Patient care**	• Limited efficacy of current drugs • Efficacy of treatment difficult to assess • Access to treatment • Vaccine development	• Clinical trials of new drug candidates • Identification and validation of biomarkers of disease progression/cure • Strategies and policies for access to treatment • Strengthening of vaccine development
**Pathogenesis**	• Poor understanding of parasite dynamics within hosts • Limited understanding of the role of parasite diversity	• Strengthening of basic research on parasite dynamics • Large-scale studies of molecular epidemiology
**Vector control**	• Consolidating achievements where primary vectors have been controlled • Designing integrated vector control interventions for secondary and intrusive vectors • Limited sustainability of massive insecticide spraying	• Political commitment to sustain vector control • Better understanding of vector ecology and adaptation to human housing • Development of novel integrated interventions

New estimates of Chagas disease burden and its epidemiological impact provide the basis for health interventions in both endemic and nonendemic countries. Indeed, Chagas disease is responsible for one of the largest disease burden in the Americas where it is endemic, with over 6 million cases. It also has the peculiarity of being one of the few NTDs to cause most of its burden in upper-middle income countries [1], including in the United States where an estimated 300,000 cases are present, and a growing number of autochthonous cases are being identified [2,3]. In nonendemic regions such as in Europe, Chagas disease is a growing concern as well, with an estimated 68,000–120,000 patients [4]. However, the identification of infected patients remains challenging in many countries, and underreporting is still a major issue.

Contrary to most, if not all, other infectious diseases, at least two serological tests are still needed for a reliable serological diagnostic of *T*. *cruzi* infection, and additional tests need to be performed in case of discordance among tests. Cases of individuals who are seronegative with conventional serological tests but seropositive with alternative tests or *T*. *cruzi* PCR-positive have been reported [5]. Part of the discordances may be attributed to the very large genetic and antigenic diversity of *T*. *cruzi*, which has been divided into seven discrete typing units (DTUs) TcI–TcVI and Tcbat [6,7]. Current serological tests are thus based on limited sets of parasite antigens and do not reflect the entire range of diversity of parasite strains and DTUs infecting humans. While efforts have been made to identify novel parasite antigens for serological diagnostic [8], an ideal test is still urgently needed [9,10]. Improved serological tests would allow a better epidemiologic surveillance for the early detection of vectorial, congenital, and oral cases, as well as for the prevention of transfusional cases.

Molecular diagnostic by PCR and quantitative PCR (qPCR) has been refined and standardized, providing key tools for the specific detection, quantification, and genotyping of *T*. *cruzi* parasites in a variety of biological and clinical samples [11–13]. Such methods are proving critical to complement serological diagnostics, patient follow-up after drug treatment, and the unraveling of parasite transmission cycles. However, the use of PCR as a diagnostic tool is still debated, as the extreme sensitivity that has been reached is prone to false positive results and, most importantly, parasite DNA may be present without infection with live parasites, as it has been observed during congenital infection in mouse models [14,15]. These issues complicate the interpretation of PCR results. On the other hand, molecular genotyping is still insufficiently sensitive, particularly for samples with very low parasite burden [16]. Thus, linking parasite genotypes and DTUs with their biological characteristics, clinical outcomes, and transmission cycles remains elusive [17], and more sensitive genotyping tests are thus critically needed.

In spite of limited options, drug treatment of infected patients has also progressed over the past ten years. Nifurtimox and benznidazole have a confirmed efficacy in the early stages of infection, particularly in children and young adults. Although more studies are needed, the treatment of women of child-bearing age may also prevent congenital transmission to their newborns [18,19]. Importantly, a pediatric formulation of benznidazole has been developed under the leadership of Drugs for Neglected Diseases Initiative (DNDi), making drug administration to children easier (Clinicaltrial.gov registration: NCT02625974). However, drug shortage, lack of registration in many countries, poor access to health care, and limited awareness of the disease in both patients and health providers all reduce access to treatment for patients. Current estimates suggest that only 1% have access to these trypanocidal drugs [20,21]. Eliminating the barriers limiting access to treatment is thus a key priority for the forthcoming years, and this will require strong political commitment.

Nonetheless, the results of the long awaited Benznidazole Evaluation for Interrupting Trypanosomiasis (BENEFIT) trial, aimed at evaluating benznidazole treatment in patients with chronic chagasic cardiomyopathy, have stressed the limitations of current treatment [22]. Indeed, while treated patients presented significant decreases in blood parasite levels, they showed no improvement in cardiac clinical outcomes [23]. In addition, the development of new drugs is progressing at a disappointing rate, and there are very few candidates in the development pipeline [21]. Posaconazole, once viewed as a very promising new option, failed to provide sustained reduction in circulating parasites in clinical trials, and other triazoles (i.e., ravuconazole) are not superior to benznidazole [24]. As an alternative, vaccine development against *T*. *cruzi* has benefited from a renewed interest [25] due to a better understanding of the delicate host–parasite immune balance, and vaccine development may lead to a valuable additional tool for Chagas disease control.

These recent clinical trials have also pointed out the need for a better understanding of *T*. *cruzi* pathogenesis and disease progression. Indeed, the assessment of blood parasite burden by qPCR or the reduction/disappearance of antibodies against the parasite are very imperfect end points to evaluate treatment effectiveness. New biomarkers of disease progression and treatment efficacy are urgently needed for a better follow-up of treated patients. Several candidate molecules have been described [26] but still require extensive clinical validation, which can only be achieved through the development of easy and affordable assays. The dynamics of parasite tissue distribution and the multiclonality of infections are also just beginning to be addressed and may lead to a better understanding of the delicate balance in the host-parasite relationship [27].

As mentioned above, vector control remains the main preventive intervention against Chagas disease in endemic countries, and it has been very effective at eliminating vectorial transmission by triatomine species that have adapted well to human housing such as *Triatoma infestans* [28]. Thus, several countries and regions have been declared free of vectorial transmission in the past decade [29]. However, many additional species of triatomines that are more sylvatic but with various degrees of intrusiveness to human habitat are now emerging as important vectors responsible for a significant transmission of *T*. *cruzi* to humans [28]. Their control with conventional indoor residual insecticide spraying is poorly effective and thus requires the development of novel integrative interventions based on triatomine ecology. Entomological surveillance and control thus need to be further strengthened and sustained to achieve better vector control.

While we have witnessed striking progress over the past ten years in our understanding and control of Chagas disease, the major challenges highlighted above remain to be addressed to reach the goals of the London declaration on NTD, which aims at eliminating this disease (among others) as a public health concern. There is no doubt that *PLOS Neglected Tropical Diseases* will continue to support this endeavor by providing a forum for the discussion of these challenges and our progress in addressing them.
